# Cementation of a Metal Dual Mobility Liner in Patients Undergoing Revision Total Hip Arthroplasty

**DOI:** 10.1016/j.artd.2023.101270

**Published:** 2023-12-03

**Authors:** Zhongming Chen, Sandeep S. Bains, Jeremy A. Dubin, Oliver C. Sax, Gregory A. Gilson, Danielle A. Jacobstein, Austin Nabet, James Nace, Ronald E. Delanois

**Affiliations:** LifeBridge Health, Sinai Hospital of Baltimore, Rubin Institute for Advanced Orthopedics, Baltimore, MD, USA

**Keywords:** Cementation, Dual mobility liner, Previously inserted, Acetabular component

## Abstract

**Background:**

Dual mobility liners for primary and revision total hip arthroplasties can lead to decreased dislocation and revision rates. However, there are a lack of data analyzing their utilization when cementing into well-fixed acetabular components in a variety of scenarios. Therefore, the purpose of this study was to examine cementation of dual mobility liners into well-fixed existing acetabular components from previous hip procedures, into new acetabular components, or directly into the acetabulum without prior implants. We specifically aimed to assess the following: (1) aseptic revision-free implant survivorship, (2) patient-reported outcomes, (3) prosthetic joint infections, dislocations, and osteolysis, (4) medical complications and readmissions, and (5) radiographic outcomes.

**Methods:**

A total of 35 patients who underwent treatment with a cemented dual mobility liner from October 1, 2014, to July 1, 2018, were identified and followed up for a mean of 4 years (range, 4-8). The primary outcome of interest was revision-free survival. The secondary outcomes included patient-reported outcome measurements, dislocations, periprosthetic infections, periprosthetic fractures, pulmonary emboli, deep vein thromboses, radiographic osteolysis, and emergency visits as well as inpatient admissions. The patient-reported outcome measurements used were the Hip Disability and Osteoarthritis Outcome Score for Joint Replacement, Short-Form 12 Health Survey Mental Component, and Short-Form 12 Health Survey Physical Component.

**Results:**

Aseptic revision-free survivorship was 93.3%, 92.3%, and 100% for previous acetabular cup, new cup, and native acetabulum, respectively. The Hip Disability and Osteoarthritis Outcome Score for Joint Replacement improved and the Short-Form 12 Physical Component improved postoperatively for all groups. Surgical complications included 3 prosthetic joint infections (1 in a new cup and 2 in native acetabula). A total of 1 patient (previous cup) had an emergency visit and inpatient readmission. Only 1 cemented dual mobility recipient (new cup) demonstrated progressive acetabular radiolucencies and all cemented dual mobility patients had no evidence of acetabular subsidence.

**Conclusions:**

Cemented dual mobility bearing liners demonstrated exceptional survivorship, low complication rates, adequate radiographic results, and improved functional outcomes when cemented into previously inserted well-fixed acetabular components, new components, or native acetabula. To the best of the authors’ knowledge, this is the first study to demonstrate success at a minimum of 4-year follow-up. These data are important to surgeons deciding on the appropriate implantation methods to use for their high-risk patients.

## Introduction

Hip dislocation remains one of the most common reasons for revision and rerevision total hip arthroplasty (THA) [1,2]. Surgical strategies to address unstable prostheses contributing to hip dislocation range from abductor reconstruction to using constrained liners and dual mobility (DM) constructs. As hip implant survival approaches 25 years, patients who have previous well-fixed acetabular components may need partial revisions, but the specific compatible liners may no longer be available. Additionally, other liners may better address patient symptoms such as dislocations and instability [3].

Multiple studies within the past 2 decades have demonstrated excellent biomechanical, clinical, and functional outcomes after cementing polyethylene liners to newly inserted acetabular components [[4], [5], [6], [7]]. More recently, studies have reported cementation of metal DM liners into newly inserted acetabular components. Wegrzyn et al. [8] has demonstrated this to be a biomechanically acceptable alternative to polyethylene. Gabor et al. [9] found low recurrent dislocation rates after cementation of monoblock DM liners in a new acetabular component. Regardless of liner material, this strategy can reduce hip dislocations by preserving bone stock, decreasing intraoperative blood loss, avoiding acetabular revision, decreasing operative time, and enabling liner exchange in situations where the compatible acetabular shell is unavailable. Another option is cementation of a liner into a well-fixed acetabular component from a previous hip procedure [10]. A recent case series assessing this strategy reports no recurrent dislocations. However, use of a metal DM liner in this instance required further evaluation given the increased predisposition to dislocation in this population.

A further examination is necessary given the success of polyethylene liners in these situations, but a lack in published data surrounding the use of metal DM liners. Therefore, the purpose of this study is to examine cementation of DM liners into well-fixed existing acetabular components from previous hip procedures, into new acetabular components, or directly into the acetabulum without prior implants. We specifically aimed to assess (1) aseptic revision-free implant survivorship, (2) patient-reported outcomes, (3) prosthetic joint infections, dislocations, and osteolysis, (4) medical complications and readmissions, and (5) radiographic outcomes.

## Material and methods

We reviewed a consecutive series of 35 patients at a single institution who underwent treatment with a cemented DM liner (Stryker Orthopedics, Mahwah, New Jersey and DePuy Synthes, Raynham, Massachusetts) into either a previously stable acetabular cup, new cup or directly into the acetabulum from October 1, 2014, to July 1, 2018. There were 3 patients (7.9%) excluded as they were lost to follow-up.

### Characteristics of cementation of modular dual mobility (MDM) liner

There were 15 liners cemented into a prior cup, 14 cemented into a new cup or cage, and 6 cemented into the native acetabulum. High viscosity cement with antibiotics was used. In regard to cups, there were 26 Zimmer Trabecular Metal Acetabular Revision System (Zimmer Biomet, Warsaw, Indiana), nonmodular revision cups, and 3 Trident II Acetabular System (Stryker, Mahwah, New Jersey) used in the study. In regard to liners, there were 34 Stryker MDM liners (1 28 mm, 3 36 mm, 6 38 mm, 17 42 mm, 2 46 mm, 3 48 mm, and 2 52 mm) and 1 DePuy Synthes liner (1 54 mm). Indications for revision THA included the following: 20 patients for complex acetabular reconstructions including augments, bone grafting, and/or cup-cage constructs, 10 patients for recurrent dislocation/instability, 5 patients for pelvic discontinuity. Of the patients who dislocated, 7 liners were cemented into a prior cup and 3 were cemented into a new cup all using Zimmer Revision System. Of the patients who had acetabular reconstructions, 8 liners were cemented into a prior cup, 6 liners were cemented into a new cup or cage, and 6 were cemented into the acetabulum. Of the patients who had pelvic discontinuity, all 5 liners were cemented into a new cup or cage. Of the 3 Trident II, 1 liner was cemented into a new cup or cage due to pelvic discontinuity, 1 liner was cemented into a new cup or cage due to acetabular reconstructions (as a result of damage of the locking mechanism from bone loss), and 1 liner was cemented into a prior cup due to acetabular reconstructions.

### Reasons for cementation DM liner

Reasons for cementing a DM liner consisted of (1) those with prior stable acetabular cup without an available polyethylene insert, (2) broken locking mechanism of a stable acetabular cup, (3) DM liner not available for a prior stable acetabular cup, or (4) younger patients with a previously stable cup without an available highly crosslinked polyethylene. A DM liner was cemented into a new cup as opposed to using an appropriate cup with an uncemented DM liner due to the incompatibility between the cup and the liner in forming a cup-cage construct with the latter method.

### Inclusion criteria

Utilization of DM liner cemented directly into the acetabulum was indicated for infected hips. Other inclusion criteria were (1) revision hip cases without indications for acetabular cup removal, (2) clinical follow-up with clinical notes, and (3) indications for DM liner such as chronic dislocations or instability. This study was given exemption status by a review board due to its retrospective nature.

### Surgical technique

**Figure 1:**
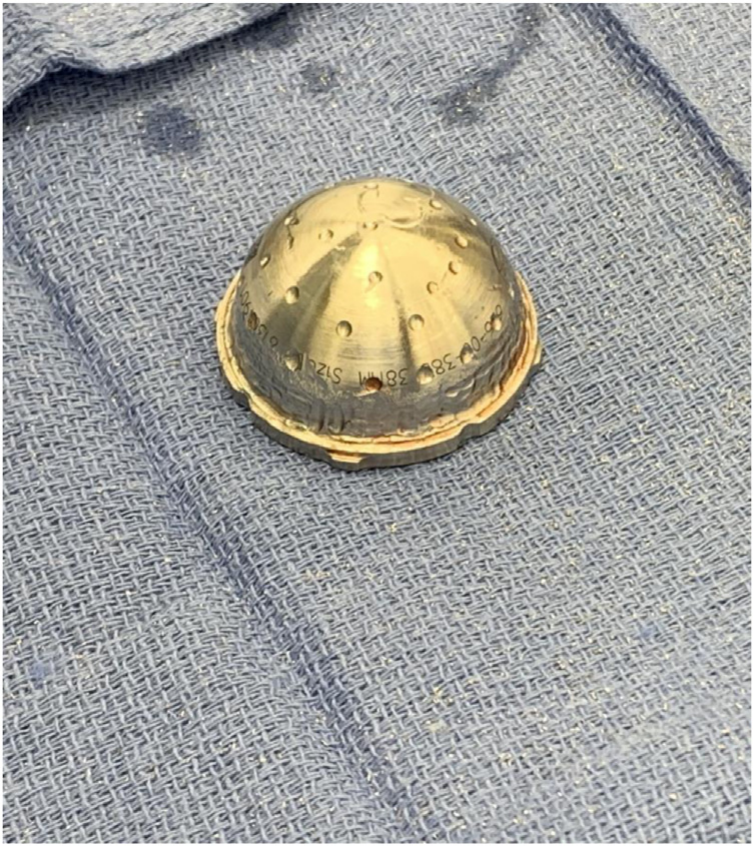
Clinical photograph of the backside of a selected metal liner.

**Figure 2:**
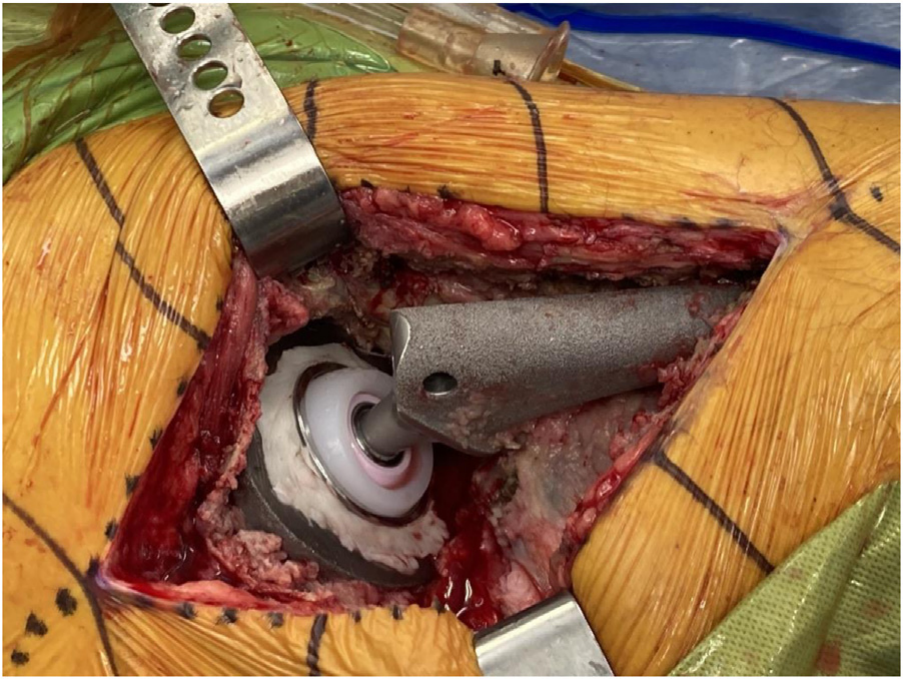
Clinical photograph of cemented metal dual mobility liner.

**Figure 3:**
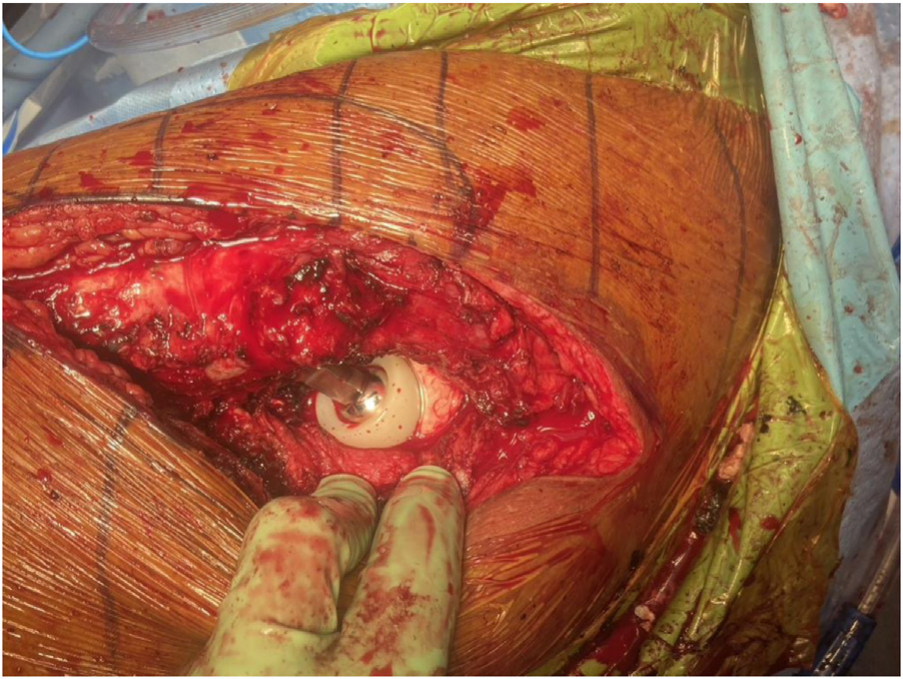
Clinical photograph of stably reduced hip.

The well-fixed acetabular component was confirmed to be well fixed. Multiple cultures were obtained. A series of trial liners were utilized to select the appropriately sized DM liner allowing for a 2- to 4-mm cement mantle. The selected metal liner was contained sufficiently within the well-fixed acetabular component in every case. The backside of the selected metal liner and the well-fixed acetabular shell were roughened with a burr to maximize metal-cement interface strength (See Fig. 1). The same pattern was used to prepare all of the liners in these cases based on our previous experience in achieving mechanical stability. Grooves were created in a circumferential pattern at a depth of 2 mm and a width of 3 mm with the use of a 4-mm burr 5 mm below the diameter of the liners. The metal liners were roughened with a high-speed drill-bit [11]. The mean burr time is 10-15 minutes. The metal DM liner was cemented within the well-fixed acetabular component with the desired version and inclination (See Fig. 2). The corresponding DM bearing and femoral head components were impacted on the femoral trunnion. The hip was reduced, trialed, and found to be stable (See Fig. 3). The patient was allowed to weight bear as tolerated postoperatively.

### Outcomes of interest

The primary outcome for this study was revision-free survival, defined as the percentage of patients that did not undergo subsequent reoperation for any cause other than infection during the study period. The secondary outcomes included patient-reported outcome measurements, dislocations, osteolysis, periprosthetic infections, periprosthetic fractures, pulmonary emboli, deep vein thrombosis, and emergency visits as well as inpatient admissions. Periprosthetic infection was defined as a deep infection of the hip determined by the Musculoskeletal Infection Society criteria. Outcomes of interest were identified individually through patient electronic records. Patient-reported outcomes were evaluated using the Hip Disability and Osteoarthritis Outcome Score, Joint Replacement, Short-Form 12 Health Survey Mental Component, and Short-Form 12 Health Survey Physical Component.

### Radiographic analyses

Radiographs were obtained at regular intervals postsurgery. Each radiograph was compared to initial postoperative radiographs and final follow-up radiographs demonstrating survival and evaluated for progressive radiolucent lines, subsidence, and failures by 2 authors (G.G and A.N). Subsidence was determined by sagittal and coronal measurement differences via operative planning software (TraumaCad, Brainlab, Munich, Germany). Component migration with >2 mm of radiolucency was the threshold for subsidence as previously described [11]. These analyses, along with documenting their respective Gruen [12], DeLee, and Charnley [13] zone locations, were performed on both cohorts.

### Patient demographics and baseline characteristics

The mean age as well as body mass index of our patient sample was 65 (range 32-92) and 28.7 (range 15-43), respectively (See Table 1). The majority of patients had a body mass index of 20-30 (62.9%). The cohort was predominantly men (60.0%), and the minority of patients had a history of alcohol (37.1%) or tobacco abuse (48.6%). The 5-item Frailty Index was 1.65 due to the greater number of hypertensive patients (51.4%) as well as a large sample needed assistance for daily living (80.0%). Most patients underwent cementing of the DM liner into a prior cup (42.9%), followed by cementing of the liner into a new cup or cage (40.0%), and cementing of the liner into the acetabulum (17.1%).

**Table 1:** Baseline patient characteristics.

Patient characteristics	n = 35	
	Total	
	n	%
Age (y)	65.11 (Range, 31.75-92.29)	
BMI (kg/m^2^)	28.7 (Range, 15-43)	
BMI <20	2	5.71%
BMI 20-30	22	62.86%
BMI 30-40	10	28.57%
BMI >40	1	2.86%
Gender		
Male	21	60.00%
Female	14	40.00%
Alcohol abuse	13	37.14%
Tobacco users	17	48.57%
5-item Frailty index	1.65	
COPD	4	11.43%
DM	4	11.43%
CHF	2	5.71%
HTN	18	51.43%
ADL	28	80.00%
HIV	0	0.00%
Hepatitis C	3	8.57%
Renal failure	3	8.57%
CKD stage ≥3		0.00%
Laterality		
Left	22	62.86%
Right	13	37.14%
Cemented into prior cup	15	42.86%
Cemented into new cup or cage	14	40.00%
Cemented into acetabulum	6	17.14%

ADL, activities of daily living; BMI, body mass index; CHF, congestive heart failure; CKD, chronic kidney disease; COPD, chronic obstructive pulmonary disease; DM, diabetes mellitus; HIV, human immunodeficiency virus; HTN, hypertension.

### Data analyses

Data were analyzed and depicted using Microsoft Excel (Microsoft, Redmond, Washington). Descriptive statistics were generated for baseline patient characteristics, medical and surgical outcomes, as well as patient-reported outcomes.

## Results

### Revision-free survivorship

Aseptic revision-free survivorship was 93.3%, 92.3%, and 100% for previous acetabular cup, new cup, and native acetabulum with a minimal follow-up of 4 years, respectively (See Table 2). There were no cases in which the cemented acetabular liners loosened. Of those that did have a revision, 1 patient was for 2 total dislocations, in which a new cup was placed. The other patient was revised for aseptic loosening, in which the liner was still well fixed in the cup at the time of the revision. Once revised, both patients did not require further reoperations and were both revised to a 42-mm MDM liner with a 70-mm Zimmer Trabecular Metal Acetabular Revision System, which was the same components before the revision. There were no subsequent rerevisions, dislocations, or complications in these patients.

**Table 2:** Medical and surgical outcomes.

	n = 35	
	Total	
	n	%
30-d ED visits	1	2.86%
30-d inpatient readmissions	1	2.86%
PE/DVT	3	8.57%
PNA	0	0.00%
Revisions	2	5.71%
Aseptic loosening	1	2.86%
Dislocations causing revision	1	2.86%
Dislocations	2	5.71%
Osteolysis	0	0.00%
PJI	3	8.57%

DVT, deep vein thrombosis; ED, emergency department; PE, pulmonary embolism; PJI, periprosthetic joint infection; PNA, pneumonia.

### Patient-reported outcome measurements

The baseline Hip Disability and Osteoarthritis Outcome Score, Joint Replacement, was 50 out of 100, while postoperatively 64 out of 100 (See Table 3), *P* < .001. The Short-Form 12 physical component had a gain of 15 (30-45), *P* < .001, whereas the mental component stayed the same (44).

**Table 3:** Patient-reported outcomes.

	n = 35	Follow up score	*P* value
	Total		
	Baseline score		
HOOS, JR	50	64	< .001
SF 12 MCS	44.44	44.11	.90
SF 12 PCS	30.47	44.88	< .001

HOOS, JR, Hip Disability and Osteoarthritis Outcome Score, Joint Replacement; MCS, Mental Component Score; PCS, Physical Component Score; SF, Short Form.

### Surgical complications

Surgical complications included 2 total dislocations; of which, both did not have any further dislocations thus not requiring subsequent revision (See Table 2). Furthermore, 3 patients did have a prosthetic joint infection requiring revision without further complications. All 3 patients had a history of tobacco use and body mass index >30 with no history of prior infections. One infection was caused by *Escherichia coli* and the other 2 were unknown.

### Medical complications and readmissions

The emergency visit and inpatient readmission was from the same patient (3%) not relating to the hip surgery (See Table 2). Of note, 3 patients (8.6%) did have either a pulmonary emboli or deep vein thromboses.

### Radiographic analyses

**Figure 4:**
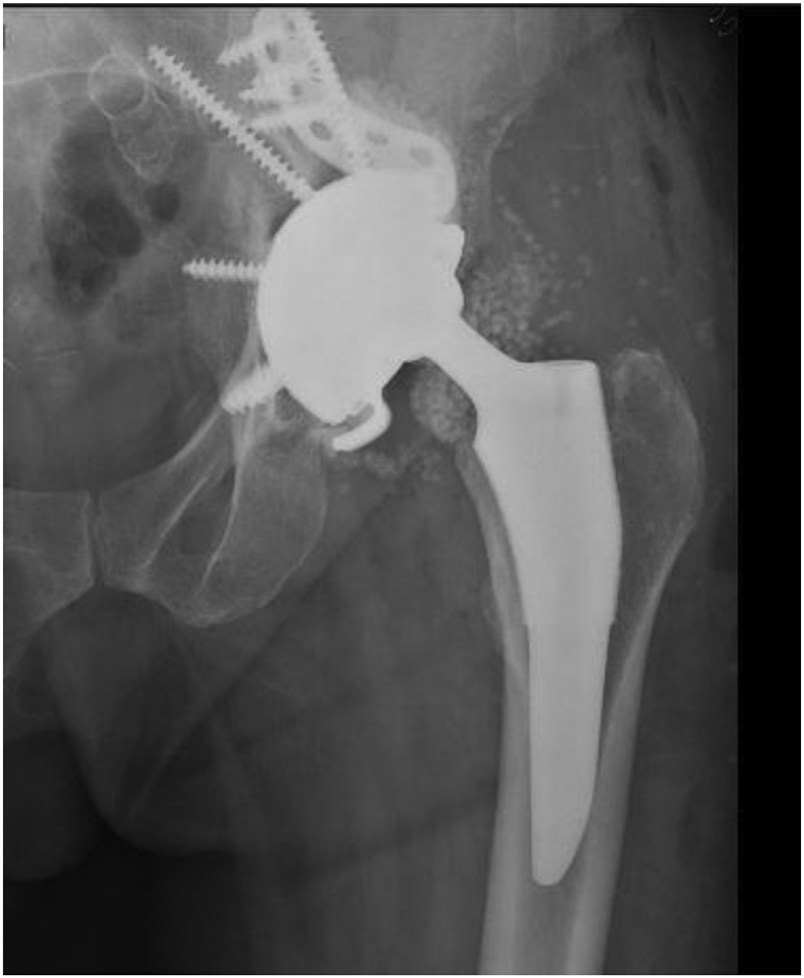
Radiograph of cup-cage construct with MDM liner.

**Figure 5:**
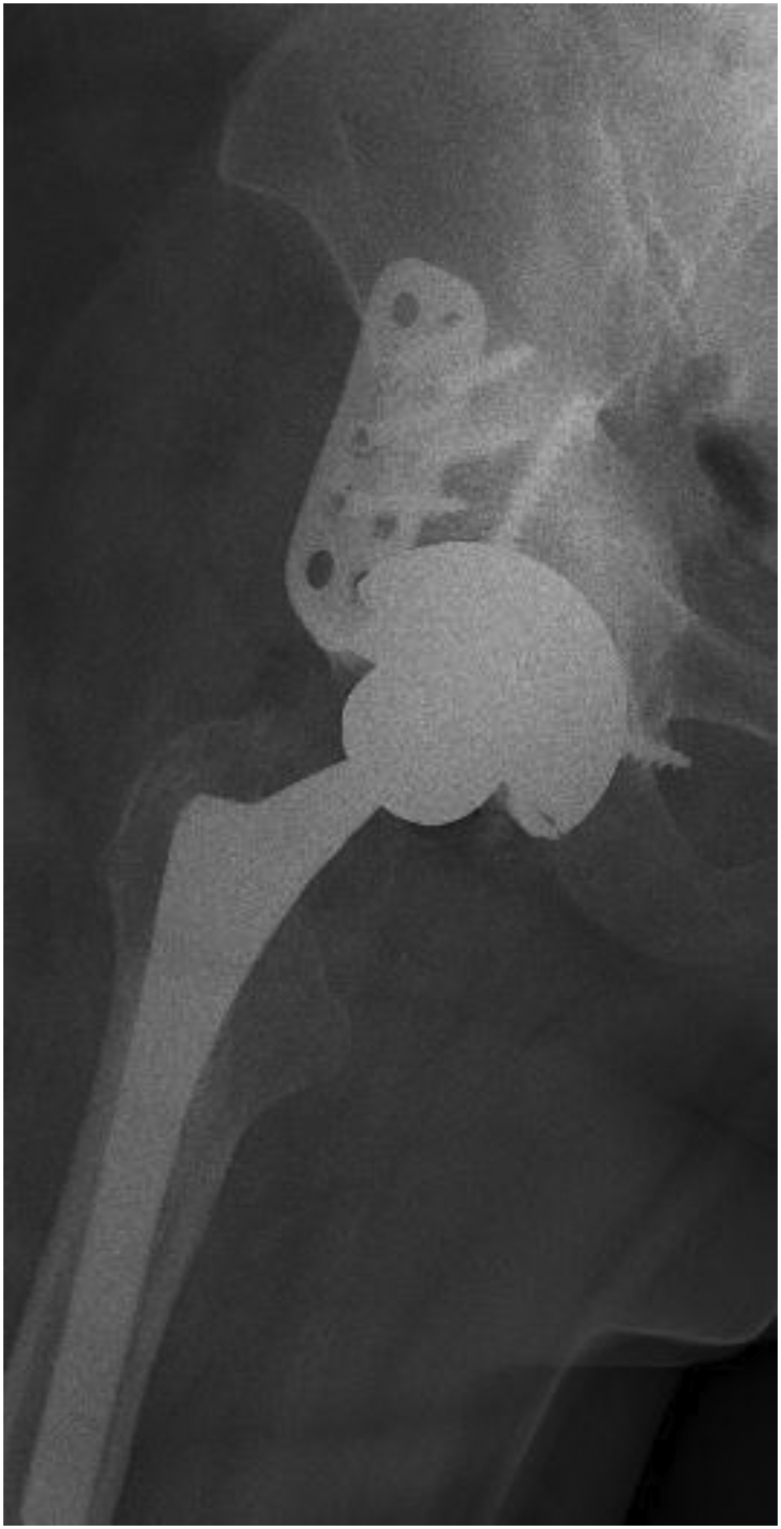
Revision for aseptic loosening of loose acetabular cup.

A total of 34 out of 35 (97.1%) cemented DM recipients did not demonstrate progressive acetabular radiolucencies. However, on the radiograph of 1 patient did show signs of acetabular loosening that led to an aseptic revision. This patient had a 42-mm MDM liner cemented into the native cup. A total of 35 out of 35 (100%) cemented DM patients did not have acetabular subsidence. We included radiographs of the cup-cage construct with MDM liner (Fig. 4) and the postoperative image of the loose acetabular cup that was revised for aseptic loosening (Fig. 5).

## Discussion

THA DM acetabular systems have been found to have decreased dislocation and revision rates [[14], [15], [16], [17], [18], [19], [20], [21], [22], [23]]. Analyses on cementing metal DM liners in the setting of a newly inserted acetabular component are lacking [[4], [5], [6], [7]]. We found that most patients did not require further revision as survivorship was 94.3% at a minimal follow-up of 4 years. For those that did not undergo rerevision, the other surgical complications included 2 dislocations. Additionally, 3 patients (8.6%) suffered from a pulmonary emboli or deep vein thromboses. Hip Disability and Osteoarthritis Outcome Score, Joint Replacement, scores demonstrated an increase from 50 preoperatively to 64 postoperatively (*P* < .001), while the Short-Form 12 physical component improved from 30 to 45 (*P* < .001). A total of 34 out of 35 (97.1%) cemented DM recipients did not demonstrate progressive acetabular radiolucencies and 35 out of 35 (100%) cemented DM patients did not have acetabular subsidence.

This study is not without limitations. This is a single-institution study without a comparison group. Additionally, due to this procedure being uncommon, a small population sample was enrolled in this study; however, these patients were followed up for a minimum of 4-years with positive outcomes. Inserting cemented liners into new acetabular components and cemented liners into well-fixed acetabular components are expected to have a different set of complications and longevities. Several studies show similar longevities and low risk of dislocation between cementation into new acetabular components and cementation into well-fixed components [9,10]. Although this may limit the generalizability of the overall findings, Chalmers et al. [24] similarly showed favorable survivorship and low rate of complications in their cohort of patients with new and well-fixed acetabular components. While we did not compare the use of cemented and cementless DM components in revision surgery, the literature supports similar complication rates and survivorship regardless of fixation type [25,26]. Operative time data would allow us how to get a sense of fast this technique is but was not available in our analysis. Despite these limitations, the authors believe that this is excellent information that was closely monitored over an extended period on this rare and novel topic.

Few studies report on exclusively cementless DM liners for use with cement. Plummer et al. [27] reported a low failure rate (11%) with no revisions for dislocation in their analysis of 36 DM liners used. However, they combined several types of DM, including MDM (Stryker), anatomic DM (Stryker) and polar cup (Smith & Nephew) in their analysis. Chalmers et al. [24] demonstrated no DM cup dissociation at the cement-cup interface in 18 patients undergoing exclusively cementation of anatomic DM components into a stable acetabular shell. To our knowledge, our study is the first to report on outcomes of exclusively cementless MDM liners for use with cementation.

Other studies support our results in demonstrating satisfactory survivorship, low complication rates, adequate radiographic results, and improved functional outcomes with cementation of metal DM liners into newly inserted well-fixed acetabular components [9,10,26]. Moreta *et al.*, reported on the results of cementing a DM component into a stable acetabular shell in 10 high-risk patients from a single institution between 2012 and 2016 undergoing revision THA with a mean follow-up of 3.5 years [26]. They found that at latest follow-up, Harris hip scores had improved from 49.3 preoperatively to 71.3 postoperatively (*P* = .098). Additionally, postoperative recurrent dislocation only occurred in 1 hip. Furthermore, they did not find any cases of intraprosthetic dislocation, aseptic loosening of the previous shell, or dissociation at the cement-cup interface. Gabor *et al.*, in a multicenter study, investigated 38 patients (38 hips) who received a cemented monoblock DM cup [9]. At a median follow-up of 216 days (range, 6-783 days), they examined 90-day complications and readmissions, revision for any reason, as well as Harris hip scores. They demonstrated that the Harris hip score improved from a mean of 50 ± 12.2 to 78 ± 11.2 (*P* < .001). Additionally, only 1 patient (2.6%) experienced a dislocation (on postoperative day 1), which was closed reduced with no further complications. Furthermore, there was also just 1 (2.6%) reoperation for periprosthetic joint infection treated successfully with a 2-stage exchange. Wegrzyn *et al.* [10] evaluated the outcome of the “double- socket” technique, cementation of a DM cup into the existing well-fixed metal shell, in revision THA for late instability. They studied 28 revision THAs (28 patients) for a mean follow-up of 3.5 years (range, 2-5 years). They reported that the mean operative time from the incision to wound dressing was 107 minutes (range, 75-140 minutes), the mean intraoperative bleeding was 200 mm (range, 110-420 mm), and no intraoperative complications. They found that the mean postoperative Harris hip scores improved significantly from 71 (range, 69-74) to 88 (range, 82-95) (*P* < .01). No postoperative complications, reoperations, or rerevisions were reported. Additionally, no dislocations, dissociations of the cemented dual mobility cup construct at the metal shell/cement interface, or aseptic loosening of the retained metal shell were observed. Therefore, the authors of these reports supported the technique of cementing a DM cup into a newly inserted metal shell as a blood-sparing procedure with reliable results.

Some suggest caution with the cementation of DM components in THAs [28]. While our results and those of the reports mentioned previously demonstrated a small risk of dislocation after these operations, John *et al.*, detail a case of complete dissociation of the cemented DM cup and metal shell from the acetabulum [25]. A patient underwent revision THA after sustaining 3 dislocations after their primary procedure and received a cemented DM liner. Postoperatively, the patient was asymptomatic for the next year when they sustained a fall in the bathroom. At presentation, they were unable to bear weight and radiographic analyses demonstrated a completely dissociated acetabular socket lying outside the acetabulum. During rerevision surgery, the DM liner and metal shell were found to be completely loose. An acetabular reinforcement cage was implanted along with a cemented cup and constraint liner. At 1-year follow-up, the patient was mobile, asymptomatic, and did not have any further episodes of instability. Therefore, the authors concluded that the use of cemented DM liners is still controversial and orthopaedic surgeons should be aware of the possibility of this complication when using metal-backed cemented DM liners. However, the authors also admit that this phenomenon is very rare.

Roughening of the convex backside of liner has been shown to improve the strength of the cement-liner interface and increase mechanical stability using a burr or sagittal saw [11,29,30]. Haft et al. [31] found that constructs with a circumferentially grooved and nubbed liner had a significantly greater yield moment and maximum moment than any other constructs. Similarly, Bonner et al. [30] showed that the creation of circumferential grooves significantly improved the strength of the constructs (*P* < .001). Lim et al. [7] corroborated that roughening of the backside of the liner using a high-speed bur in a circumferential pattern to a depth of 2 mm ensures good cement interdigitation and providing lever-out as well as torsional stability. This pattern of grooves is consistent, even in commercially available DM cups that are designed to be cemented.

## Conclusions

DM bearing liners demonstrate exceptional survivorship, low complication rates, adequate radiographic results, and improved functional outcomes. To the best of our knowledge, this is the first study to demonstrate success at minimum 4-year follow-up. These data are important to surgeons deciding on the appropriate implantation methods to use for their high-risk patients.

## Conflicts of interest

Ronald Delanois received research support from Johnson & Johnson, Biocomposites, CyMedica Orthopedics, Depuy Synthes Product, Flexion Therapeutics, Microport Orthopedics, Orthofix, Patient-Centered Outcomes Research Institute (PCORI), Smith & Nephew, Stryker, Tissue Gene, and United Orthopedic Corporation; is a part of Journal of Knee Surgery editorial board; and is a board member of the Baltimore City Medical Society. James Nace is a board member of Arthritis Foundation; is a part of editorial board for Journal of Arthroplasty, Journal of the American Osteopathic Medicine Association, Orthopedic Knowledge, and Journal of Knee Surgery; received research support from Microport Orthopedics, Stryker, and United Orthopedic Corporation; and is a paid consultant for Microport Orthopedics. All other authors declare no potential conflicts of interest.

For full disclosure statements refer to https://doi.org/10.1016/j.artd.2023.101270.

## Appendix A Supplementary data

Conflict of Interest Statement for Nabet

Conflict of Interest Statement for Jacobstein

Conflict of Interest Statement for Gilson

Conflict of Interest Statement for Dubin

Conflict of Interest Statement for Nace

Conflict of Interest Statement for Sax

Conflict of Interest Statement for Delanois

Conflict of Interest Statement for Bains

Conflict of Interest Statement for Chen
