# Supplementary material for: Cementation of a Metal Dual Mobility Liner in Patients Undergoing Revision Total Hip Arthroplasty
Source: Arthroplast Today. 2023 Dec 3;24:101270. doi: 10.1016/j.artd.2023.101270 (PMC10704222; doi:10.1016/j.artd.2023.101270)
Supplement: Conflict of Interest Statement for Delanois [file mmc7.docx]

# INDIVIDUAL CONFLICT OF INTEREST STATEMENT

***American Association of Hip and Knee Surgeons***

(Adopted from the American Academy of Orthopaedic Surgeons disclosure statement)

The following form **must be filled out completely and submitted by each author (example, 6 authors, 6 forms).**

**All items require a response. If there is no relevant disclosure for a given item, enter "*None*.”**

**Manuscript Title**

1. Royalties from a company or supplier (The following conflicts were disclosed)

N/A

2. Speakers bureau/paid presentations for a company or supplier (The following conflicts were disclosed)

N/A

3A. Paid employee for a company or supplier (The following conflicts were disclosed)

N/A

3B. Paid consultant for a company or supplier (The following conflicts were disclosed)

N/A

3C. Unpaid consultants for a company or supplier (The following conflicts were disclosed)

N/A

4. Stock or stock options in a company or supplier (The following conflicts were disclosed)

N/A

5. Research support from a company or supplier as a Principal Investigator (The following conflicts were disclosed)

Johnson & Johnson

Biocomposites

CyMedica Orthopedics

Depuy Synthes Product

Flexion Therapeutics

Microport Orthopedics

Orthofix

Patient-Centered Outcomes Research Institute (PCORI)

Smith & Nephew

Stryker

Tissue Gene

United Orthopedic Corporation

6. Other financial or material support from a company or supplier (The following conflicts were disclosed)

N/A

7. Royalties, financial or material support from publishers (The following conflicts were disclosed)

N/A

8. Medical/Orthopaedic publications editorial/governing board (The following conflicts were disclosed)

Journal of Knee Surgery

9. Board member/committee appointments for a society (The following conflicts were disclosed)

Baltimore City Medical Society

**Each author must sign AND print or type his/her name, date and submit a separate form**

In addition, one BLINDED Conflict of Interest form (no author names used) should be submitted per manuscript with all author disclosures.

Ronald Delanois Ronald Delanois 9/1/22

Author Name (Print or Type) Author Signature Date
